# Comprehensive Profiling of the Rice *OsEPF/EPFL* Gene Family Under Biotic and Abiotic Stresses Reveals the Involvement of *OsEPF2* in Disease Resistance

**DOI:** 10.3390/plants15172657

**Published:** 2026-08-30

**Authors:** Mingliang Guo, Yingying Tang, Tianhao Liu, Zeyuan She, Di Wang, Xianghui Meng, Dagang Tian, Yuan Qin

**Affiliations:** 1 College of Life Sciences, College of Agriculture, Fujian Provincial Key Laboratory of Haixia Applied Plant Systems Biology, Haixia Institute of Science and Technology, Fujian Agriculture and Forestry University, Fuzhou 350002, China; gml604@163.com (M.G.);; 2 Fujian Provincial Key Laboratory of Genetic Engineering for Agriculture, Biotechnology Research Institute, Fujian Academy of Agricultural Sciences, Fuzhou 350003, China; 3 State Key Laboratory for Conservation and Utilization of Subtropical Agro-Bioresources, Guangxi Key Lab of Sugarcane Biology, College of Agriculture, Guangxi University, Nanning 530004, China

**Keywords:** rice, EPIDERMAL PATTERNING FACTOR, biotic/abiotic stresses, broad-spectrum, disease resistance

## Abstract

Members of the EPIDERMAL PATTERNING FACTOR (EPF) and EPF-Like (EPFL) families perform diverse regulatory functions in plant tissue morphogenesis, controlling the development of stomata, awns, shoot apical meristems (SAMs), and inflorescences. Nevertheless, the biological functions of *OsEPF/EPFL* family members in mediating responses to biotic/abiotic stresses are not yet widely characterized. Here, we demonstrated abundant *cis*-acting elements in the putative promoters of *OsEPF/EPFL* genes, including those associated with dehydration-, MeJA-, MYB binding site for drought, stress-, and ABA-responsive element. We performed a systematic analysis of the expression patterns of all *OsEPF/EPFL* family members under heat, cold, drought, and salt stress treatments. Among these genes, *OsEPFL9* and *OsEPFL10* exhibited rapid and sustained up-regulation across all four stress conditions. Furthermore, the majority of *OsEPF/EPFL* members were up-regulated specifically in response to salt stress. In terms of biotic stress responses, *OsEPF2/5/7/10* were rapidly induced as early as 12 h post-infection (hpi) with the rice blast pathogen (*Magnaporthe oryzae*). Functional validation further revealed that its deficiency causes increased sensitivity to both *M. oryzae* and *Xanthomonas oryzae* pv. *oryzae* (*Xoo*). Collectively, our research will provide significant insights into the multifunctional roles of the *OsEPF/EPFL* gene family, particularly in stress responses. This work also establishes a theoretical basis and scientific reference for the application of plant small secreted peptides (SSPs) in crop disease resistance breeding and stress tolerance improvement.

## 1. Introduction

Cell-to-cell communication coordinates environmental and developmental responses [1]. Small signaling peptides (SSPs), typically composed of 2–100 amino acid residues, are classified into secreted and non-secreted types based on their N-terminal sequences. Non-secreted peptides remain intracellular and exert their functions within the cell, whereas secreted peptides are synthesized intracellularly and subsequently transported to the extracellular space. Secreted peptide signals are classified into two main categories: post-translationally modified small peptides and cysteine-rich peptides (CRPs) [2]. Some of these secreted peptides can even move long distances via the xylem and phloem, mediating intercellular communication across different tissues and organs [3].

To date, the best-characterized small secreted peptides (SSPs) are those involved in plant development processes, with the main families including systemin (Sys) [4], ROOT GROWTH FACTOR (RGF) [5], CLAVATA3 (CLV3) [6], CLAVATA3/EMBRYO-SURROUNDING REGION (CLE) [7], C-TERMINALLY ENCODED PEPTIDE (CEP) [8], rapid alkalinization factor (RALF) [9], S-LOCUS CYSTEINE-RICH PROTEIN (SCR/SP11) [10], phytosulfokine (PSK) [11], EPIDERMAL PATTERNING FACTOR (EPF) [12], and the EPIDERMAL PATTERNING FACTOR-LIKE (EPFL) [13] family, etc. EPIDERMAL PATTERNING FACTOR/EPF-LIKE (EPF/EPFL) is a unique class of cysteine-rich small secreted peptides in plants. In recent years, EPF/EPFL family genes have been well characterized. For example, a total of 11 EPF/EPFL family members were identified in *Arabidopsis* [14], 12 in rice [15], 18 in maize [16], 12 in sorghum [17], 33 in oat [18], 11 in barley [19], 27 in oilseed rape [20], and 135 in cotton [21].

Multiple EPF/EPFL family proteins have been functionally characterized. Genes of this family govern diverse core developmental events covering leaf, stomatal, seed, awn, embryo and panicle formation, and are also vital regulators of drought and cold stress tolerance in plants. Several members of the EPF/EPFL family have been demonstrated to regulate stomatal development in *Arabidopsis thaliana* [14,22,23]. *AtEPF1*, *AtEPF2*, *AtEPFL5*, and *CHALLAH* (*AtEPFL6*) act as negative regulators of stomatal development in *Arabidopsis*, whereas *AtEPFL9* functions as a positive regulator [24,25,26]. The EPF/EPFLs also play roles in other processes, such as filament elongation, fertility, and stamen identity [27,28]. In rice, *GRAIN NUMBER*, *GRAIN LENGTH AND AWN DEVELOPMENT1* (*GAD1*)/*REGULATOR OF AWN ELONGATION 2* (*RAE2*)/*GRAIN LENGTH AND AWN DEVELOPMENT* (*GLA*) encodes an EPF/EPFL family peptide that mediates awn development and regulates grain length and grain number, with these agronomic functions thought to be selected during rice domestication [1,29,30]. *OsEPFL10* positively regulates stomatal density, and *osepfl10* exhibited reduced stomatal density, yet sustained photosynthetic rates and displayed improved water-use efficiency [31].

While the EPF/EPFL gene family is well-established as a key regulator of various organs in plants, and its contributions to growth and development are increasingly understood, the function of most family members remains poorly characterized. However, although the functions of some EPF/EPFL family genes have been reported, research on their role in regulating plant stress resistance is still very limited. Therefore, having valuable insights into the structure, function, and mechanism of action of EPF/EPFL genes can provide novel strategies for crop genetic improvement, thereby enhancing resistance to various environmental stresses and improving crop yield and quality. Here, we investigated the *cis*-acting elements within the putative promoter regions of *OsEPF/EPFL* family members and comprehensively analyzed the responsiveness of *OsEPF/EPFL* family genes to biotic stress (*M. oryzae* and *Xoo* infection) and abiotic stresses (heat, cold, drought, and salt treatments). Moreover, this study revealed that small peptides *OsEPF2* regulated disease resistance in rice, and the above results will provide a theoretical basis and scientific reference for the application of plant SSPs in crop production and breeding.

## 2. Results

### 2.1. Characterization of Stress- and Hormone-Related cis-Acting Elements in OsEPF/EPFL Gene Promoters

**Figure 1:**
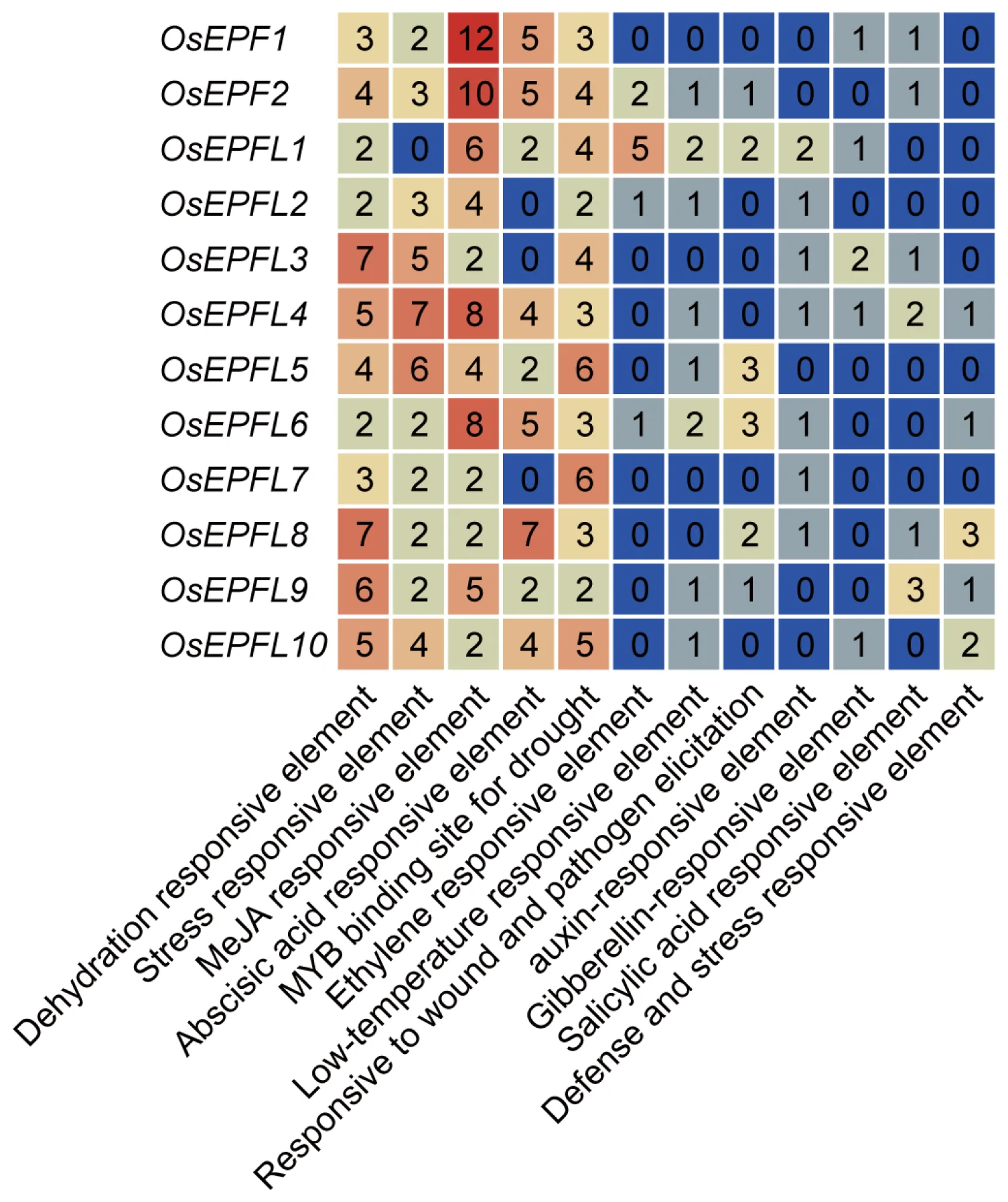
*Cis*-acting element analysis of *OsEPF/EPFL* genes associated with environmental stress and hormone responses. Heatmap showing the number of diverse environmental stress- and hormone-related *cis*-elements identified within the 2000 bp upstream promoter sequences of *OsEPF/OsEPFL* genes. Rows represent individual genes, and columns represent distinct *cis*-element types. Each cell represents the count of corresponding *cis*-responsive elements. High copy numbers are indicated in red, whereas zero copies are shown in dark blue. A red-to-blue color gradient represents high-to-low abundance.

The *cis*-acting promoter elements direct the coordinated transcription of their target genes; therefore, dissecting these regulatory sequences is essential to link observed expression patterns to the underlying genetic circuitry. The putative promoter sequences (about 2 kb upstream) of the *OsEPF/EPFL* genes were extracted from the Phytozome database and the *cis*-acting elements associated with stress responses were identified by PlantCARE [32]. In the putative promoter regions, 296 *cis*-acting elements were observed, which could be classified into 12 types related to stress and hormone responses (Figure S1; Tables S4 and S5). The number of *cis*-acting elements are presented in Figure 1. The results display that *OsEPF1* and *OsEPF2* contain a high number of MeJA-responsive elements. The distribution is presented in Figure S1 and Table S5. These *cis*-elements were located on both the sense (+) and antisense (−) strands of double-stranded DNA. Different colors correspond to different functional categories of *cis*-elements, including phytohormone-, abiotic- and biotic-stress-related motifs (Figure S1). Dehydration-responsive element, MeJA-responsive element, MYB binding site for drought, Stress-responsive element, and Abscisic acid-responsive element were widely distributed in the promoter regions of most genes. These findings suggest that *OsEPF/EPFL* genes might participate in drought-stress responses and tolerance, with promoter *cis*-acting elements that mirror and support *EPF/EPFL* gene family predicted functions.

### 2.2. Transcriptional Responses of OsEPF/EPFL Genes Under Simulated Abiotic Stress Conditions

In rice, *OsEPFL10* knockout decreases stomatal density, enhances water retention, and consequently elevates drought tolerance [31]. Whereas the *EPF/EPFL* gene family is well documented in drought and low-temperature stress in many plants, its involvement in other abiotic stresses remains largely unexplored. Abundant environmental stress-related and hormone-responsive elements are present in the *OsEPF/EPFL* promoter regions (Figure 1); therefore, how do these genes respond to abiotic stresses?

**Figure 2:**
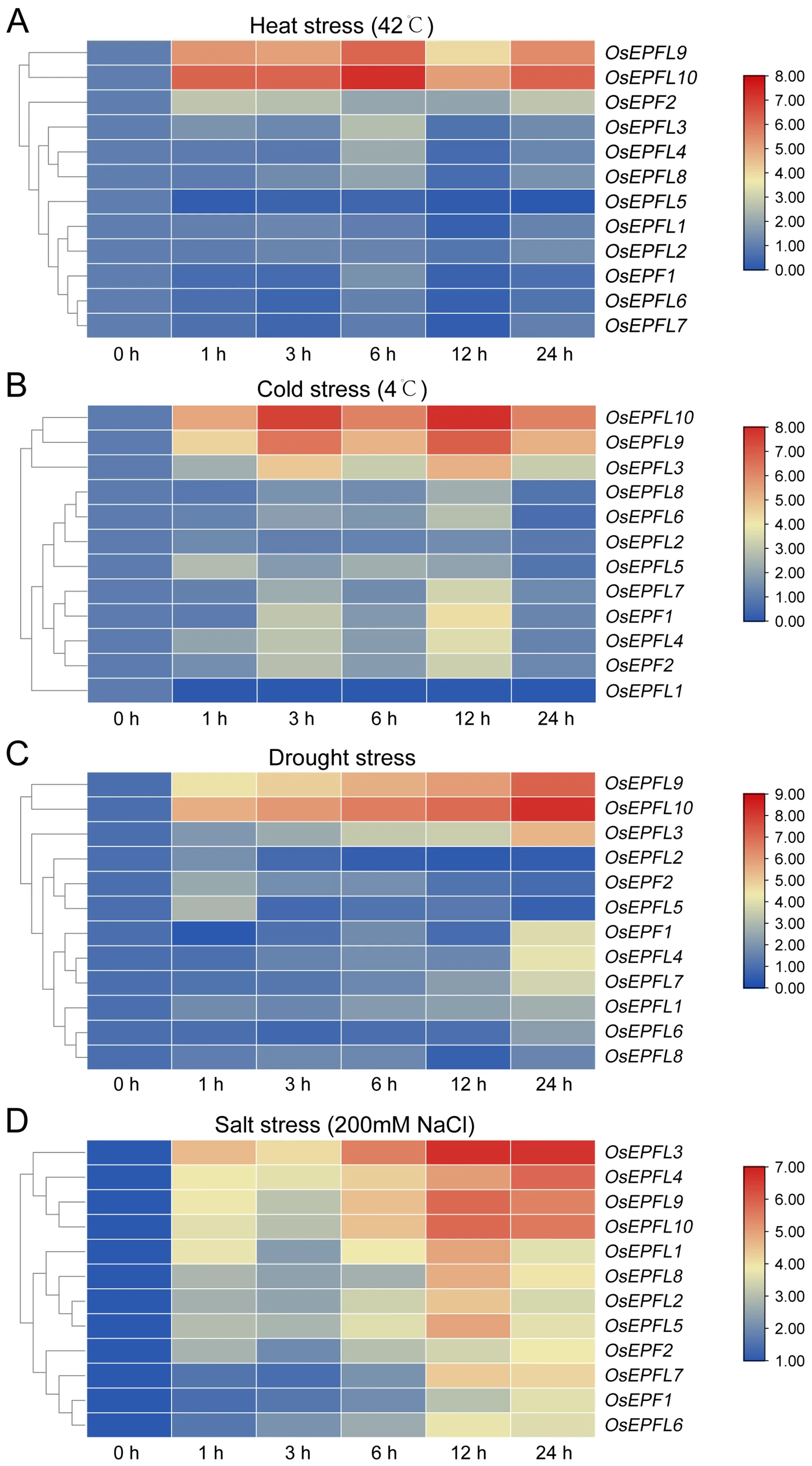
Relative expression level of *OsEPF/EPFL* genes under abiotic stress. Temporal expression profiles of *OsEPF/EPFL* genes under heat (**A**), cold (**B**), drought (**C**), and salt (**D**) stress treatments were generated by qRT-PCR. The qRT-PCR values of each *OsEPF/EPFL* gene, normalized to the reference gene *OsUBQ5*, were set to 1 in the unstressed sample (0 hpi) using the 2^−ΔΔCT^ method. Three independent biological replicates were performed for each sample (n = 3). The heatmap was generated using the TBtools software. Rows correspond to genes and columns to time points. A blue-to-red color gradient represents low-to-high relative expression.

To explore the potential roles of *OsEPF/EPFL* genes under simulated abiotic stresses, we analyzed the expression patterns of 12 family members in rice seedlings exposed to heat (42 °C), cold (4 °C), drought, and salt treatments by qRT-PCR. It is worth noting that *OsEPFL9* and *OsEPFL10* exhibited rapid and sustained transcriptional up-regulation under heat, cold, drought, and salt treatments (Figure 2). In addition, *OsEPF2* responded quickly to heat stress (Figure 2A), and *OsEPFL5* was rapidly up-regulated under both cold and drought treatments (Figure 2B,C). Notably, the majority of *OsEPF/EPFL* members were transcriptionally up-regulated in response to salt stress (Figure 2D). Taken together, the above results demonstrated that *OsEPF/EPFL* family members displayed both functional conservation and differentiation in abiotic stress responses, offering a solid theoretical framework for dissecting their roles and underlying mechanisms.

### 2.3. Expression of OsEPF/EPFL Genes Under M. oryzae Stress

Previous investigations of the *OsEPF/EPFL* family have concentrated on its roles in growth, development, and abiotic stresses such as drought and low temperature, while its involvement in biotic stress remains largely unexplored, especially under pathogen attack.

**Figure 3:**
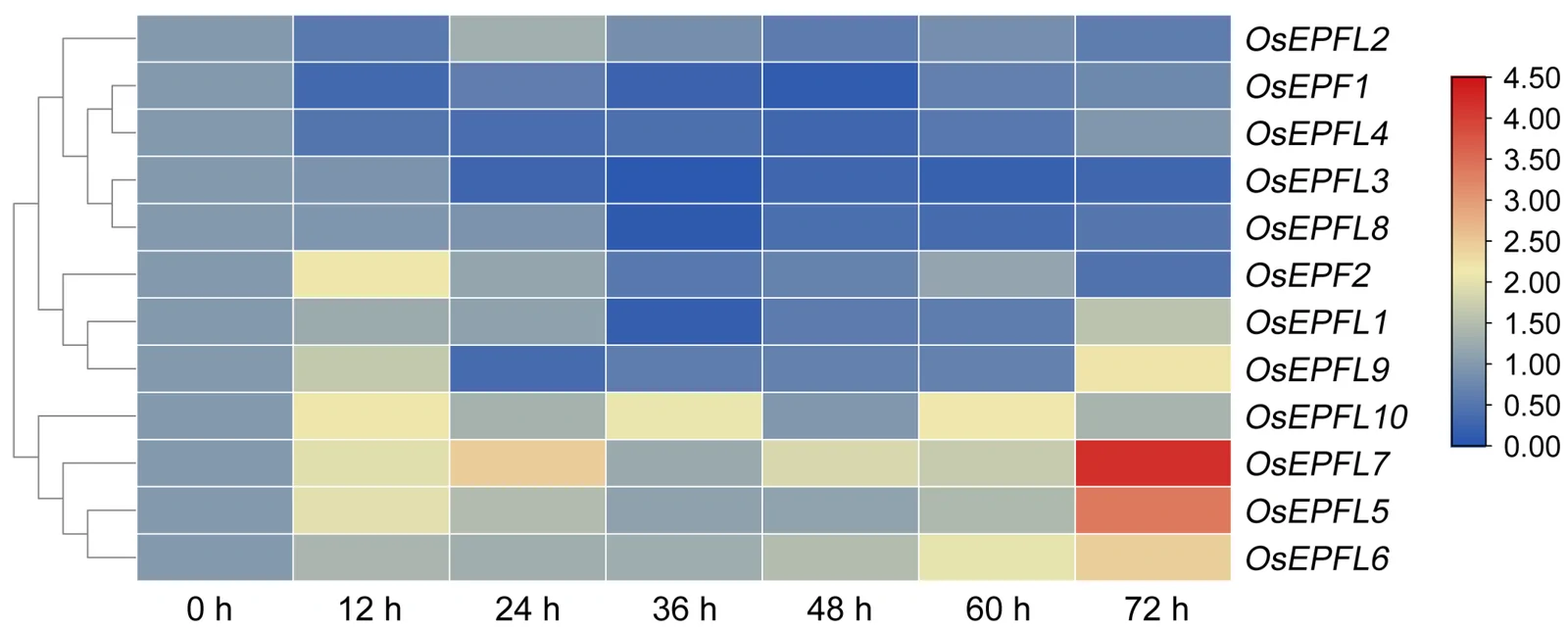
Expression analysis of *OsEPF/EPFL* genes under *Magnaporthe oryzae* stress. To characterize the response of *OsEPF/EPFL* genes to rice blast pathogen (*M. oryzae*) infection, the temporal expression patterns were generated using qRT-PCR. The qRT-PCR values of each *OsEPF/EPFL* gene, normalized to the reference gene *OsUBQ5*, were set to 1 in the unstressed sample (0 hpi) using the 2^−ΔΔCT^ method. Three independent biological replicates were performed for each sample (n = 3). The heatmap was generated using the TBtools software. Rows correspond to genes and columns to time points. A blue-to-red color gradient represents low-to-high relative expression.

To comprehensively understand the response of *OsEPF/EPFL* genes to biotic stress, we analyzed the profiling of all members following challenge with the blast fungus using qRT-PCR (Figure 3). The results exhibited that *OsEPF2* was specifically up-regulated at 12 hpi (hours post inoculation), while *OsEPFL5*, *OsEPFL7*, and *OsEPFL10* were likewise induced at this time point, suggesting that these four genes might quickly sense and response to pathogen infection to further modulate innate immunity.

### 2.4. OsEPF2 Positively Regulates Disease Resistance in Rice

**Figure 4:**
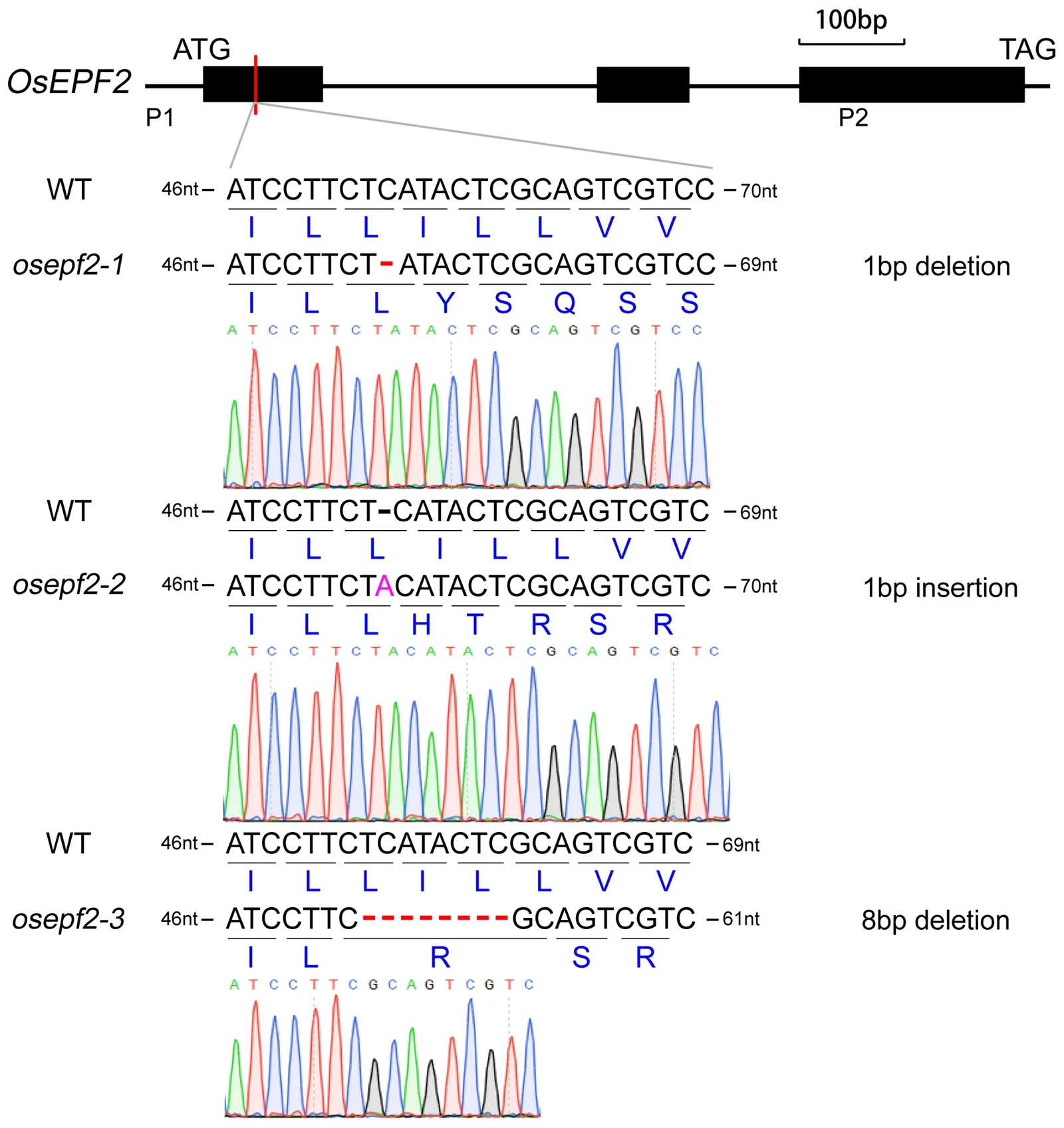
Genotyping analysis of *osepf2*. The gene structure of *OsEPF2* is shown at the top. Three CRISPR/Cas9-mediated mutations in *OsEPF2* are identified with P1/P2 primers in the T2 generation. The diagrams show mutation sequences in *OsEPF2*, with black letters indicating base pairs matching WT in the target site. The pink letters indicate inserted base pairs in the mutants; red dotted lines indicate nucleotide deletions; and dark blue letters indicate amino acids encoded by WT and *osepf2* sequences.

The above results showed that *OsEPF2* was specifically up-regulated at 12 hpi (Figure 3). Then, what about the function of *OsEPF2*? To determine whether *OsEPF2* was involved in stress resistance, we constructed *osepf2* mutants using the CRISPR/Cas9 method with single guide RNAs (sgRNA). Three homozygous mutant lines containing different mutations in the first exon of *OsEPF2* were obtained in the T2 generation through the PCR and sequencing using the primer listed in Table S1. As shown in Figure 4, three mutant strains carried frameshift mutations: *osepf2-1* contained a one-base-pair deletion, *osepf2-2* a one-base-pair insertion, and *osepf2-3* an eight-base-pair deletion. The qRT-PCR analysis revealed that the expression level of *OsEPF2* in CRISPR-edited lines was significantly lower than that in ZH11 (Figure S2), with the primers listed in Table S2.

**Figure 5:**
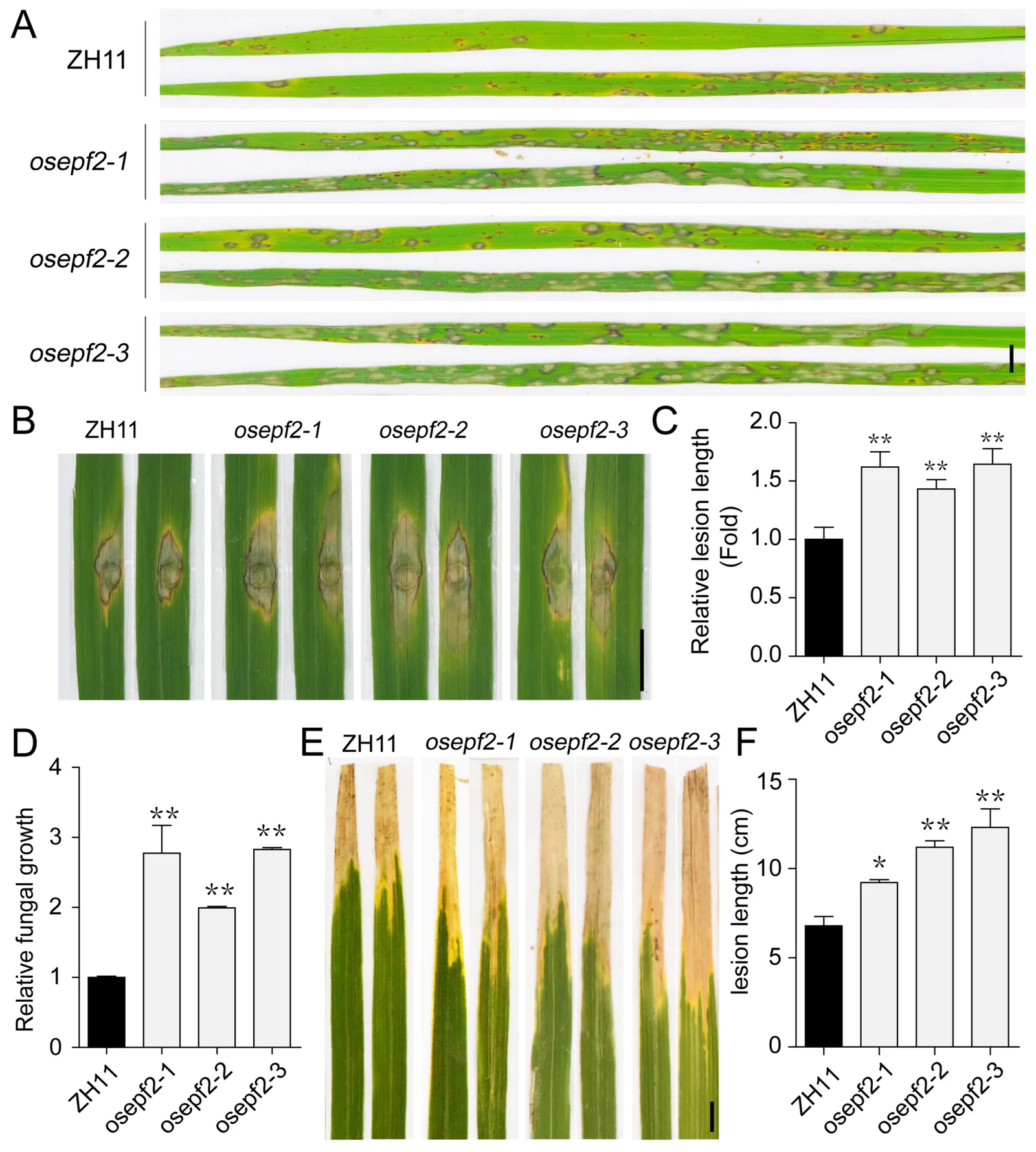
*OsEPF2* positively regulates disease resistance in rice. (**A**) Evaluation of the rice blast resistance of the *osepf2* via the spray method. Three-week-old *osepf2* and ZH11 plants were sprayed with the *M. oryzae*-isolated strain Guy11. The diseased lesions were observed 5 days after inoculation. Bar = 5 mm. (**B**) Evaluation of the rice blast resistance of the *osepf2* via the punch method. One-month-old *osepf2* and ZH11 plants were inoculated with the *M. oryzae*-isolated strain Guy11 by the punch method, and after 8 d inoculation, diseased lesions were observed. Bar = 1 cm. (**C**) The relative lesion length in *osepf2* compared to ZH11 after *M. oryzae* inoculation. (**D**) Assessment of the relative fungal growth in diseased *osepf2* and ZH11 leaves via RT-qPCR at the DNA level. Total DNA was extracted from the diseased leaves, and relative fungal growth was detected by RT-qPCR using the primers listed in Table S3. (**E**) Analysis of the response of *osepf2* plants to *Xoo* infection. Leaves of *osepf2* plants at two weeks after *Xoo* inoculation. Bar = 1 cm. (**F**) The statistics of lesion length in *osepf2* compared to ZH11 after *Xoo* inoculation. Black vertical lines indicate the scale bar in (**A**,**B**,**E**). Data are given as means ± SD. Asterisks (* *p* < 0.1; ** *p* < 0.01 by Student’s *t*-test) represent significant differences between the ZH11 and *osepf2*.

To investigate the effects of *osepf2* function on biotic stress resistance, three-week-old seedlings of *osepf2-1/2/3* and wild-type ZH11 were sprayed with the *M. oryzae*-isolated strain Guy11. ZH11 is a widely used *japonica* rice accession. For rice blast resistance, ZH11 carries the functional blast resistance locus *Pizh* (*Pizh-1/Pizh-2*), but lacks several well-known broad-spectrum blast resistance genes including *Pigm*, *Pi9*, and *Pi2/Piz-t* [33,34]. After inoculation for five days, wild-type ZH11 exhibits moderate susceptibility to Guy11, and *osepf2* lines exhibited more disease lesions than ZH11 (Figure 5A). To further validate the results, one-month-old plants of *osepf2-1/2/3* and ZH11 were inoculated with Guy11 using the punch method (Figure 5B). Seven days post-inoculation with *M. oryzae*, the relative lesion lengths of *osepf2-1/2/3* were larger than ZH11 (Figure 5C). To assess the relative fungal growth in diseased *osepf2* and ZH11 leaves, total DNA was extracted from the diseased leaves and the fungal biomass of *osepf2-1/2/3* leaves were greater than that in the ZH11 (Figure 5D) via RT-qPCR using the primers listed in Table S3. For bacterial blight resistance, ZH11 does not harbor major dominant *Xa* resistance genes such as *Xa1*, *Xa4*, and *Xa21*, and is susceptible to multiple compatible *Xoo* strains [35]. To further characterize the role of *OsEPF2* in biotic stress, an inoculation assay with *Xoo* strains P6 (PXO99) showed that ZH11 displayed susceptibility to the *Xoo* strain, and *osepf2-1/2/3* lines exhibited markedly increased susceptibility, displaying significantly longer lesions than the ZH11 wild type (Figure 5E,F). Collectively, these results imply that *OsEPF2* participates in the regulation of disease resistance in rice.

### 2.5. The Influence of OsEPF2 on the Expression of Defense-Related Genes

Based on the above results, *OsEPF2* regulates disease resistance in rice. As an epidermal patterning factor and a small cysteine-rich signaling peptide, OsEPF2 is capable of perceiving and recognizing signal molecules, thereby further activating a series of downstream biological processes. Then, how does it regulate defense-related genes during the initiation of plant disease resistance mechanisms?

**Figure 6:**
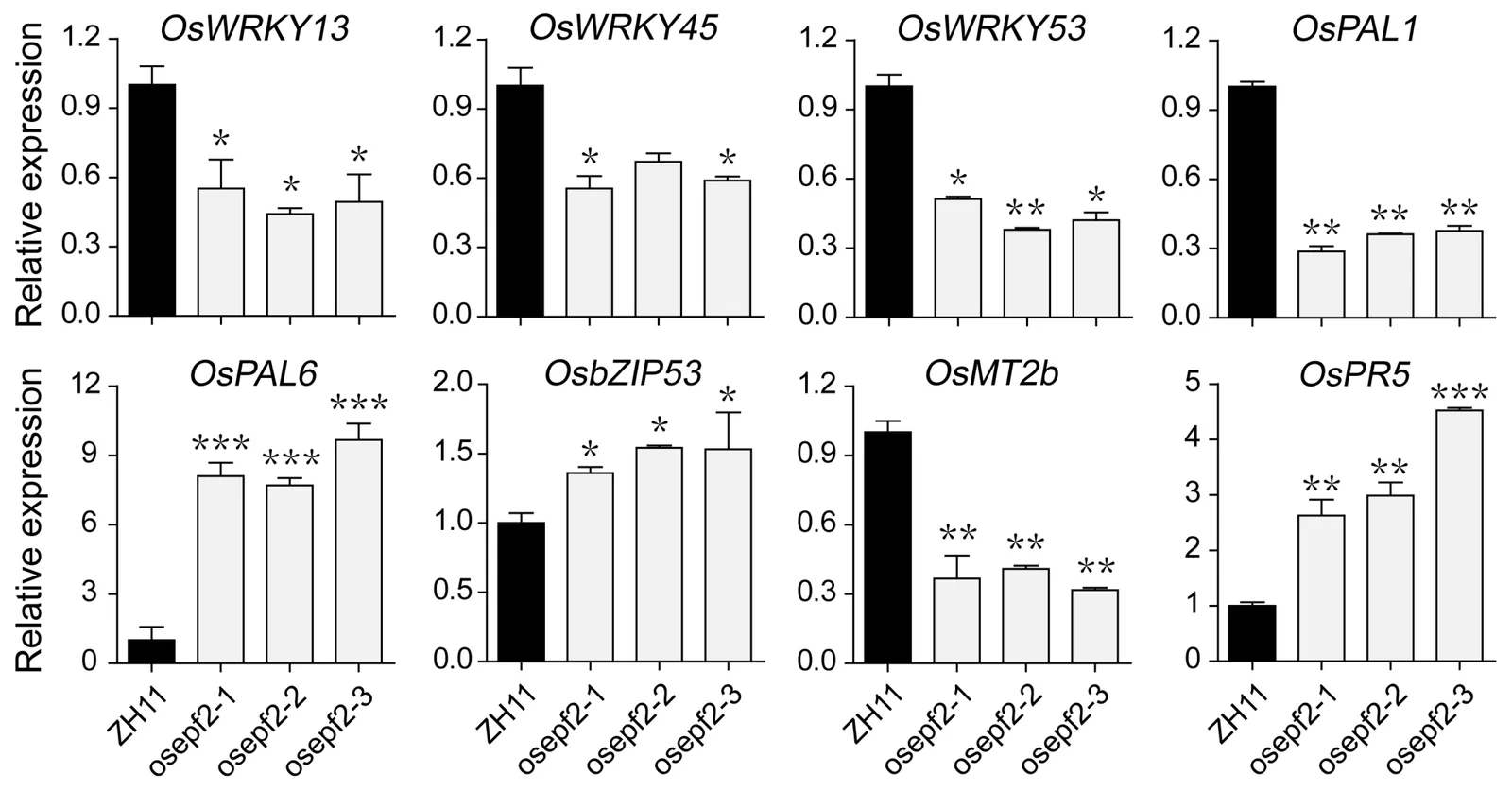
Determining the expression of defense-related genes in *osepf2* and wild-type ZH11 after *M. oryzae* inoculation. Three-week-old *osepf2* and wild-type ZH11 plants were sprayed with the *M. oryzae* isolated strain Guy11. Samples were collected at 24 h after *M. oryzae* inoculation, and the expression levels of defense-related genes in these samples were determined via qRT-PCR. Data are given as means ± SD. Asterisks (* *p* < 0.05; ** *p* < 0.01; *** *p* < 0.001 by Student’s *t*-test) represent significant differences between the ZH11 and *osepf2*.

Thus far, numerous defense genes have been cloned and thoroughly characterized in rice, such as *OsWRKY13*, *OsWRKY45*, *OsWRKY53*, *OsPAL1*, *OsPAL6*, *OsbZIP53*, *OsMT2b*, and *OsPR5* [36,37,38,39,40,41,42,43]. To analyze the expression levels of defense-related genes in *osepf2*, leaf samples were collected from three-week-old *osepf2* and ZH11 plants at 24 hpi following spray inoculation with *M. oryzae* strain Guy11 by qRT-PCR using the primers listed in Table S6. The results showed that *OsWRKY13*, *OsWRKY45*, *OsWRKY53*, *OsPAL1*, and *OsMT2b* were significantly down-regulated in three *OsEPF2* mutant lines compared with wild-type ZH11 via qRT-PCR (Figure 6). Reversely, *OsPAL6*, *OsbZIP53*, and *OsPR5* were obviously up-regulated in *osepf2*. These observations suggested that the loss-of-function of *OsEPF2* can alter the transcriptional expression of defense-related genes, which further indicates that *OsEPF2* displays an important role in disease resistance in rice. However, the regulatory mechanism underlying the interaction between *OsEPF2* and resistance-related genes requires further investigation.

## 3. Discussion

Plants express more than a thousand small secreted peptides (SSPs) that exhibit remarkable structural diversity, undergo distinct post-translational modifications, and engage specific receptors to mediate precise physiological functions, thereby orchestrating plant–environment interactions. Recent peptidogenomic studies in the model plant *Arabidopsis* have cataloged more than 2000 conventional peptides (CPs) and over 1000 non-conventional peptides (NCPs), underscoring their fundamental roles in plant biology [44]. Since 1991, some plant peptide families have been discovered, when an 18-amino-acid peptide controlling disease and pest resistance was first identified in tomato [45].

The EPIDERMAL PATTERNING FACTOR/EPF-like (EPF/EPFL) gene family encodes plant-specific secretory peptides. Small secreted peptides regulate gene expression and participate in essential biological processes including development and defense. The *EPF/EPFL* gene family primarily confers drought stress tolerance by reducing stomatal density, which in turn lowers transpiration rates. In barley, *HvEPF1* enhances drought tolerance by lowering stomatal density and improving water-use efficiency [19]. Similarly, overexpression of *AtEPF2* achieves higher water-use efficiency without compromising photosynthetic capacity, resulting in greater drought resistance in *Arabidopsis* [46]. *PdEPF2*, *PdEPFL6*, *MdEPF2*, *Bna.EPF2*, *TaEPF1*, and *OsEPFL10* all influence plant drought resistance by regulating stomatal density [31,47,48,49]. When the *cis*-elements in the promoters of *OsEPF/EPFL* family genes were analyzed, it was observed that these promoters harbored a high abundance of *cis*-acting elements, specifically the Dehydration-responsive element, MeJA-responsive element, Drought-associated MYB binding site, Stress-responsive element, and Abscisic acid-responsive element (Figure 1). These findings suggest that *OsEPF/EPFL* genes may be involved in water-use efficiency, drought-stress response and tolerance, and their promoter *cis*-acting elements align with and support the predicted functions of the EPF/EPFL gene family. Differential composition of stress and hormone-related *cis*-elements in promoters provides a plausible molecular explanation for the divergent transcriptional responses of *OsEPF/EPFL* genes upon biotic and abiotic stresses. This study presents a systematic and comprehensive analysis of how every member of the *OsEPF/EPFL* gene family responds to heat, cold, drought, and salt stress treatments. For example, *OsEPF2*, *OsEPFL3*, *OsEPFL9* and *OsEPFL10* rapidly respond to heat, cold, drought and salt stresses. Consistently, multiple Dehydration-responsive elements, Stress-responsive elements, Abscisic acid-responsive elements and MYB transcription factor binding sites are distributed in the promoter regions of these genes (Figure 2 and Figure 3).

Notably, *OsEPFL9* and *OsEPFL10* exhibited rapid and sustained up-regulation under these treatments (Figure 2 and Figure 3). These results establish a foundation for identifying key *OsEPF/EPFL* family genes that mediate abiotic-stress responses and expand the dynamic expression atlas of this gene family under adverse conditions.

The stomata on plant leaves function as key channels for photosynthesis, transpiration, and gas exchange. Their developmental processes, including quantity, distribution, and opening–closing characteristics, are dynamically and closely associated with plant disease resistance. Stomata function not only as crucial gateways for pathogen entry but also as key signaling sites that trigger the initiation of plant immune defense. Although the *OsEPF/EPFL* family’s involvement in biotic stress has been little explored, our study presents a comprehensive expression profile of these genes in response to rice blast infection. Notably, the results demonstrated that *OsEPF2* was specifically up-regulated at 12 hpi (hours post inoculation), with *OsEPFL5*, *OsEPFL7*, and *OsEPFL10* likewise induced at this time point (Figure 3). Furthermore, we generated an *OsEPF2* mutant using CRISPR-Cas9 technology, and subsequent assays demonstrated that *OsEPF2* plays a role in conferring resistance to both rice blast and bacterial leaf blight (Figure 4 and Figure 5). Given the current data, additional functional tests employing *OsEPF2*-overexpressing transgenic lines are required to corroborate the positive regulatory effect of *OsEPF2* on disease resistance.

In addition, the loss-of-function of *OsEPF2* can alter the transcriptional expression of defense-related genes (Figure 6). OsEPF/EPFL signaling peptides could perceive external cues and relay signals to downstream cascades, presumably via the ERECTA family. ERf leucine-rich repeat receptor kinases are established cell-surface receptors for EPF/EPFL peptides, triggering intracellular MAP kinase cascades to modulate plant growth, development and stress responses [50,51]. Loss-of-function of *OsEPF2* significantly changes the expression of *OsWRKY13*, *OsWRKY45*, *OsWRKY53*, *OsPAL1*, *OsPAL6*, *OsbZIP53*, *OsMT2b* and *OsPR5*. Rather than directly modulating these genes, *OsEPF2* may engage in mutual feedback regulation with WRKY and bZIP transcription factors. In *Arabidopsis*, the *EPF1*/*EPF2* genes reduce stomatal density by inhibiting stomatal differentiation [22,52]. Specifically, research has demonstrated that overexpression of *EPF2* in *Arabidopsis* decreases stomatal density by 40% and significantly enhances resistance to the leaf spot pathogen (*Pseudomonas syringae*) [53,54]. Although our RT-qPCR results demonstrated significant transcriptional induction of *OsEPF2* under cold, heat, drought and salt treatments, phenotypic assessments of abiotic stress tolerance were not conducted using *OsEPF2* knockout lines in this study. The primary focus of the current research was to characterize the disease resistance mediated by *OsEPF2* against rice pathogens, and thus biotic stress phenotypic analyses were prioritized. The stress-responsive *cis*-elements distributed in the *OsEPF2* promoter together with its stress-inducible expression collectively suggest its potential participation in abiotic stress adaptation; however, definitive phenotypic evidence remains lacking. Further systematic abiotic stress tolerance tests employing both knockout and *OsEPF2*-overexpressing transgenic plants will be implemented in our subsequent investigations to fully uncover the dual regulatory functions of *OsEPF2* in biotic and abiotic stress responses. As is known, *EPF/EPFL* genes regulate stomatal density; we ask whether *OsEPF2*-mediated resistance operates through altered stomatal density and, consequently, through stomatal immunity that triggers broader plant defenses.

## 4. Materials and Methods

### 4.1. Plant Materials and Growth Condition

Plant materials, including the mutant lines of *OsEPF2* and wild-type Zhonghua 11 (ZH11, *Oryza sativa* L.), were cultivated in a greenhouse maintained at 22~32 °C and 60–70% humidity under a 14 h light/10 h dark photoperiod. To investigate *OsEPF2* functions, single-gene knockout mutants were generated via CRISPR/Cas9 gene editing using 20 bp gRNAs (5′ TCTCATACTCGCAGTCGTCC 3′) [55,56]. The recombinant vector was then introduced into ZH11 through Agrobacterium-mediated transformation. To check for mutations at the *OsEPF2* target site, all T2 transgenic plants were sequenced using P1/P2 primers listed in Table S1.

Two-week-old seedlings were treated at 42 and 4 °C for heat and cold stresses, respectively. Drought, salt stress (200 mM NaCl), and *M. oryzae* treatments were performed as previously described [57,58]. Two-week-old seedlings were sprayed with a spore suspension (1 × 10^5^ spores/mL) of the *Magnaporthe oryzae* (*M. oryzae*) isolate Guy11. Samples from abiotic stresses (heat, cold, drought, and salt) were collected at 0, 1, 3, 6, 12, and 24 h after the corresponding treatments, and leaf samples of biotic stresses (*M. oryzae*) were harvested at 0, 12, 24, 36, 48, 60, and 72 h post-inoculation (hpi). All samples were collected in triplicate, immediately snap-frozen in liquid nitrogen, and stored at −80 °C for RNA extraction.

### 4.2. RNA Isolation and qRT-PCR Analysis

The RNeasy Plant Mini Kit (Qiagen, Hilden, Germany) was used to extract total RNA from all collected samples, and the PrimeScript RT-PCR Kit (Takara, Shiga, Japan) was used for reverse transcription following the manufacturer’s instructions [59]. The qRT-PCR was performed on a Bio-Rad system (Foster City, CA, USA) as previously described [60]. The rice *OsUBQ5* gene was used as an internal control [61]. The comparative 2^−ΔΔCT^ method was used to determine the relative expression levels of the tested genes [62,63], and the specific primers are listed in Table S2.

### 4.3. Cis-Element Analysis in OsEPF/EPFL Gene Promoter Regions

The promoter (about 2 kb regions upstream of the start codons) of all 12 family members were extracted from Phytozome (https://phytozome-next.jgi.doe.gov/, accessed on 7 June 2021) [64]. The *cis*-elements involved in biotic and abiotic stress responses were analyzed using PlantCARE (http://bioinformatics.psb.ugent.be/webtools/plantcare/html/, accessed on 22 February 2025), as previously described [32].

### 4.4. Heatmap Analysis of OsEPF/EPFL Members

The heatmaps were generated based on the relative expression values of *OsEPF/EPFL* genes determined by qRT-PCR under abiotic (heat, cold, drought and salt) and biotic (*M. oryzae*) treatments. The qRT-PCR values of each *OsEPF/EPFL* gene, normalized to the reference gene *OsUBQ5*, was set to 1 in the unstressed sample (0 hpi) using the 2^−ΔΔCT^ method. Three independent biological replicates were performed for each sample (n = 3). Visualization of the heatmaps was done using TBtools-II v2.466 as previously described [58,65,66].

### 4.5. Rice Blast Fungus Punch Inoculation

For punch inoculation, leaves of 4-week-old rice seedlings were wounded with a mouse-ear punch, and 10 µL of spore suspension (5 × 10^5^ spores mL^−1^) was applied to each wound site as previously described [67]. After inoculation, plants were incubated for 24 h at 26 °C in a dark, humid chamber, then transferred to a humid growth room at 26 °C with a 12 h light/12 h dark photoperiod. Disease phenotypes were assessed and photographed at 6~8 days post-inoculation [68]. Following punch inoculation, diseased leaf samples were harvested, affixed to double-sided adhesive tape, scanned with a scanner, and lesion lengths were quantified using Image J (v1.43U) software. To assess the fungal biomass of *M. oryzae* in rice leaf tissues, RT-qPCR was performed using the *Pot2* gene as a molecular marker [69]. The primers in this experiment are presented in Table S3.

### 4.6. Xoo Inoculation Assay in Rice Leaves

Leaf-clipping inoculation was performed as previously described [70]. Briefly, *Xanthomonas oryzae* pv. *oryzae* (*Xoo*) strains PXO99 were cultured in LB medium at 28 °C for 48 h suspended in 10 mM MgCl_2_ to OD_600_ = 0.5, and the top 2–3 cm of fully expanded leaves at the tillering stage were cut with bacteria-dipped scissors. Disease symptoms were assessed two weeks after inoculation, quantified by measuring lesion length [71].

## 5. Conclusions

Plant growth and development are orchestrated by complex, multigene regulatory networks. The present study focuses on the roles of EPIDERMAL PATTERNING FACTOR/EPF-LIKE (EPF/EPFL) family genes in mediating plant responses to biotic and abiotic stresses. Specifically, we enriched the dynamic expression profiles of rice *OsEPF/EPFL* family genes under stress conditions and elucidated a key function of *OsEPF2*: its role in regulating disease resistance in rice. Notably, as a peptide ligand, the specific receptors of OsEPF2 in the regulation of disease resistance, as well as the detailed molecular mechanisms underlying this regulatory process, remain to be further investigated. In summary, a deeper understanding of the roles of *EPF/EPFL* genes in plant growth, development, and biotic/abiotic stress responses, along with their application in agricultural production, is highly significant for enhancing crop stress resistance and increasing yields.

## Data Availability

All relevant data are provided in Supplementary Figures S1 and S2 and Tables S1–S6.

## Supplementary Materials

The following supporting information can be downloaded at: https://www.mdpi.com/article/10.3390/plants15172657/s1, Figure S1: *Cis*-acting element distribution (quantity, type, and position) in the putative promoters of *OsEPF/EPFL* genes. Schematic representation of *cis*-element positions within the 2000 bp sequences upstream of the translation start site of *OsEPF/OsEPFL* genes. The horizontal bar represents each promoter fragment from −2000 bp (5′) to 0 bp (3′). Triangles indicate the location of distinct *cis*-responsive elements; upward triangles represent sense-strand (+) elements, and downward triangles represent antisense-strand (−) elements. Different colors correspond to different functional categories of *cis*-elements, including phytohormone-, abiotic- and biotic-stress-related motifs. Figure S2: Relative expression levels of *OsEPF2* in three mutant lines. Data are given as means ± SD. ** *p* < 0.01 compared with wild-type ZH11 using Student’s *t*-test. Table S1: Primers used for genotyping analysis of *osepf2*. Table S2: Primers used in this study for expression profiling by qRT-PCR. Table S3: Primers used for fungal biomass analysis. Table S4: Classification of *cis*-acting elements in the putative promoter regions of *OsEPF/EPFL* genes. Table S5: Number of environmental stress-related and hormone-responsive *cis*-acting elements in *OsEPF/EPFL* genes. Table S6: Primers used in this study for defense-related gene assays by qRT-PCR. Sequence data in this study can be found in the Rice Genome Annotation Project data libraries under the following accession numbers: *OsUBQ5* (*LOC_Os01g22490*); *OsEPF1 (LOC_Os04g38470)*; *OsEPF2 (LOC_Os04g54490)*; *OsEPFL1 (LOC_Os08g37890)*; *OsEPFL2 (LOC_Os02g51950)*; *OsEPFL3 (LOC_Os03g51660)*; *OsEPFL4 (LOC_Os03g46930)*; *OsEPFL5 (LOC_Os07g04020)*; *OsEPFL6 (LOC_Os03g06610)*; *OsEPFL7 (LOC_Os11g37190)*; *OsEPFL8 (LOC_Os05g39880)*; *OsEPFL9 (LOC_Os01g60900)*; *OsEPFL10 (LOC_Os01g68598).*

## References

[1] Jin J., Hua L., Zhu Z., Tan L., Zhao X., Zhang W., Liu F., Fu Y., Cai H., Sun X. (2016). *GAD1* Encodes a Secreted Peptide That Regulates Grain Number, Grain Length, and Awn Development in Rice Domestication. Plant Cell.

[2] Matsubayashi Y. (2010). Post-Translational Modifications in Secreted Peptide Hormones in Plants. Plant Cell Physiol..

[3] Matsubayashi Y. (2014). Posttranslationally Modified Small-Peptide Signals in Plants. Annu. Rev. Plant Biol..

[4] Zhang H., Zhang H., Lin J. (2020). Systemin-mediated long-distance systemic defense responses. New Phytol..

[5] Matsuzaki Y., Ogawa-Ohnishi M., Mori A., Matsubayashi Y. (2010). Secreted peptide signals required for maintenance of root stem cell niche in Arabidopsis. Science.

[6] Hirakawa Y. (2021). CLAVATA3, a plant peptide controlling stem cell fate in the meristem. Peptides.

[7] Fletcher J.C. (2020). Recent Advances in *Arabidopsis* CLE Peptide Signaling. Trends Plant Sci..

[8] Aggarwal S., Kumar A., Jain M., Sudan J., Singh K., Kumari S., Mustafiz A. (2020). C-terminally encoded peptides (CEPs) are potential mediators of abiotic stress response in plants. Physiol. Mol. Biol. Plants Int. J. Funct. Plant Biol..

[9] Zhong S., Li L., Wang Z., Ge Z., Li Q., Bleckmann A., Wang J., Song Z., Shi Y., Liu T. (2022). RALF peptide signaling controls the polytubey block in *Arabidopsis*. Science.

[10] McCubbin A.G., Kao T. (2000). Molecular recognition and response in pollen and pistil interactions. Annu. Rev. Cell Dev. Biol..

[11] Sauter M. (2015). Phytosulfokine peptide signalling. J. Exp. Bot..

[12] Takata N., Yokota K., Ohki S., Mori M., Taniguchi T., Kurita M. (2013). Evolutionary relationship and structural characterization of the *EPF/EPFL* gene family. PLoS ONE.

[13] Kosentka P.Z., Overholt A., Maradiaga R., Mitoubsi O., Shpak E.D. (2019). EPFL Signals in the Boundary Region of the SAM Restrict Its Size and Promote Leaf Initiation. Plant Physiol..

[14] Hara K., Yokoo T., Kajita R., Onishi T., Yahata S., Peterson K.M., Torii K.U., Kakimoto T. (2009). Epidermal cell density is autoregulated via a secretory peptide, EPIDERMAL PATTERNING FACTOR 2 in *Arabidopsis* leaves. Plant Cell Physiol..

[15] Xiong L., Huang Y., Liu Z., Li C., Yu H., Shahid M.Q., Lin Y., Qiao X., Xiao J., Gray J.E. (2022). Small EPIDERMAL PATTERNING FACTOR-LIKE2 peptides regulate awn development in rice. Plant Physiol..

[16] Liu R., Xu K., Li Y., Zhao W., Ji H., Lei X., Ma T., Ye J., Zhang J., Du H. (2024). Investigation on the Potential Functions of *ZmEPF/EPFL* Family Members in Response to Abiotic Stress in Maize. Int. J. Mol. Sci..

[17] Jiao Z., Wang J., Shi Y., Wang Z., Zhang J., Du Q., Liu B., Jia X., Niu J., Gu C. (2023). Genome-Wide Identification and Analysis of the *EPF* Gene Family in *Sorghum bicolor* (L.) Moench. Plants.

[18] Jiang Q., Ma L., Guo S., Zhou X., Zhang Z., Cui Y., Tang J., Yi D., Wang X. (2025). Comprehensive genome-wide identification and expression analysis of the *EPF/EPFL* gene family in oat. BMC Genom..

[19] Hughes J., Hepworth C., Dutton C., Dunn J.A., Hunt L., Stephens J., Waugh R., Cameron D.D., Gray J.E. (2017). Reducing Stomatal Density in Barley Improves Drought Tolerance without Impacting on Yield. Plant Physiol..

[20] Wang S., Wang W., Chen J., Wan H., Zhao H., Liu X., Dai X., Zeng C., Xu D. (2024). Comprehensive Identification and Expression Profiling of Epidermal Pattern Factor (EPF) Gene Family in Oilseed Rape (*Brassica napus* L.) under Salt Stress. Genes.

[21] He S., Sun H., Chen Q., Yang Y., Zhou Z., Chang S., Lu S., Liang Z., Yang J., Fei X. (2025). Comprehensive identification of cotton EPF/EPFL receptors and functional characterization of the GhEPFL1-1-GhER1 module in drought tolerance. BMC Plant Biol..

[22] Hunt L., Gray J.E. (2009). The signaling peptide EPF2 controls asymmetric cell divisions during stomatal development. Curr. Biol..

[23] Lee J.S., Hnilova M., Maes M., Lin Y.C., Putarjunan A., Han S.K., Avila J., Torii K.U. (2015). Competitive binding of antagonistic peptides fine-tunes stomatal patterning. Nature.

[24] Shimada T., Sugano S.S., Hara-Nishimura I. (2011). Positive and negative peptide signals control stomatal density. Cell. Mol. Life Sci..

[25] Niwa T., Kondo T., Nishizawa M., Kajita R., Kakimoto T., Ishiguro S. (2013). EPIDERMAL PATTERNING FACTOR LIKE5 peptide represses stomatal development by inhibiting meristemoid maintenance in Arabidopsis thaliana. Biosci. Biotechnol. Biochem..

[26] Abrash E.B., Bergmann D.C. (2010). Regional specification of stomatal production by the putative ligand CHALLAH. Development.

[27] Huang Y., Tao Z., Liu Q., Wang X., Yu J., Liu G., Wang H. (2014). *BnEPFL6*, an EPIDERMAL PATTERNING FACTOR-LIKE (EPFL) secreted peptide gene, is required for filament elongation in *Brassica napus*. Plant Mol. Biol..

[28] Sun Q., Qu J., Yu Y., Yang Z., Wei S., Wu Y., Yang J., Peng Z. (2019). *TaEPFL1*, an EPIDERMAL PATTERNING FACTOR-LIKE (EPFL) secreted peptide gene, is required for stamen development in wheat. Genetica.

[29] Bessho-Uehara K., Yamagata Y., Takashi T., Makino T., Yasui H., Yoshimura A., Ashikari M. (2021). Exploring the Loci Responsible for Awn Development in Rice through Comparative Analysis of All AA Genome Species. Plants.

[30] Zhang Y., Zhang Z., Sun X., Zhu X., Li B., Li J., Guo H., Chen C., Pan Y., Liang Y. (2019). Natural alleles of *GLA* for grain length and awn development were differently domesticated in rice subspecies japonica and indica. Plant Biotechnol. J..

[31] Karavolias N.G., Patel-Tupper D., Seong K., Tjahjadi M., Gueorguieva G.A., Tanaka J., Gallegos Cruz A., Lieberman S., Litvak L., Dahlbeck D. (2023). Paralog editing tunes rice stomatal density to maintain photosynthesis and improve drought tolerance. Plant Physiol..

[32] Lescot M., Déhais P., Thijs G., Marchal K., Moreau Y., Van de Peer Y., Rouzé P., Rombauts S. (2002). PlantCARE, a database of plant cis-acting regulatory elements and a portal to tools for in silico analysis of promoter sequences. Nucleic Acids Res..

[33] Xie Z., Yan B., Shou J., Tang J., Wang X., Zhai K., Liu J., Li Q., Luo M., Deng Y. (2019). A nucleotide-binding site-leucine-rich repeat receptor pair confers broad-spectrum disease resistance through physical association in rice. Philos. Trans. R. Soc. Lond. Ser. B Biol. Sci..

[34] Wang Z., Zhong G., Zhang B., Xie Y., Gan Y., Tang D., Wang W. (2026). Rice blast pathogen effector AvrPib compromises disease resistance by targeting Raf-like protein kinase OsMAPKKK72 to inhibit MAPK signaling. J. Integr. Plant Biol..

[35] Zeng X., Luo Y., Vu N.T.Q., Shen S., Xia K., Zhang M. (2020). CRISPR/Cas9-mediated mutation of *OsSWEET14* in rice cv. Zhonghua11 confers resistance to Xanthomonas oryzae pv. oryzae without yield penalty. BMC Plant Biol..

[36] Cheng H., Liu H., Deng Y., Xiao J., Li X., Wang S. (2015). The WRKY45-2 WRKY13 WRKY42 transcriptional regulatory cascade is required for rice resistance to fungal pathogen. Plant Physiol..

[37] Ichimaru K., Yamaguchi K., Harada K., Nishio Y., Hori M., Ishikawa K., Inoue H., Shigeta S., Inoue K., Shimada K. (2022). Cooperative regulation of *PBI1* and MAPKs controls *WRKY45* transcription factor in rice immunity. Nat. Commun..

[38] Xie W., Ke Y., Cao J., Wang S., Yuan M. (2021). Knock out of transcription factor *WRKY53* thickens sclerenchyma cell walls, confers bacterial blight resistance. Plant Physiol..

[39] Zhou X., Liao H., Chern M., Yin J., Chen Y., Wang J., Zhu X., Chen Z., Yuan C., Zhao W. (2018). Loss of function of a rice TPR-domain RNA-binding protein confers broad-spectrum disease resistance. Proc. Natl. Acad. Sci. USA.

[40] Tonnessen B.W., Manosalva P., Lang J.M., Baraoidan M., Bordeos A., Mauleon R., Oard J., Hulbert S., Leung H., Leach J.E. (2015). Rice phenylalanine ammonia-lyase gene *OsPAL4* is associated with broad spectrum disease resistance. Plant Mol. Biol..

[41] Lijuan W., Cong H., Huimei W., Yuchang H., Hai L., Lei W., Chen C., Zhiguo E. (2024). *OsbZIP53* Negatively Regulates Immunity Response by Involving in Reactive Oxygen Species and Salicylic Acid Metabolism in Rice. Rice Sci..

[42] Wong H.L., Sakamoto T., Kawasaki T., Umemura K., Shimamoto K. (2004). Down-regulation of metallothionein, a reactive oxygen scavenger, by the small GTPase OsRac1 in rice. Plant Physiol..

[43] Wang H., Meng J., Peng X., Tang X., Zhou P., Xiang J., Deng X. (2015). Rice *WRKY4* acts as a transcriptional activator mediating defense responses toward Rhizoctonia solani, the causing agent of rice sheath blight. Plant Mol. Biol..

[44] Wang S., Tian L., Liu H., Li X., Zhang J., Chen X., Jia X., Zheng X., Wu S., Chen Y. (2020). Large-Scale Discovery of Non-conventional Peptides in Maize and Arabidopsis through an Integrated Peptidogenomic Pipeline. Mol. Plant.

[45] Pearce G., Strydom D., Johnson S., Ryan C.A. (1991). A polypeptide from tomato leaves induces wound-inducible proteinase inhibitor proteins. Science.

[46] Franks P.J., Doheny-Adams T.W., Britton-Harper Z.J., Gray J.E. (2015). Increasing water-use efficiency directly through genetic manipulation of stomatal density. New Phytol..

[47] Wang C., Liu S., Dong Y., Zhao Y., Geng A., Xia X., Yin W. (2016). *PdEPF1* regulates water-use efficiency and drought tolerance by modulating stomatal density in poplar. Plant Biotechnol. J..

[48] Jiao P., Liang Y., Chen S., Yuan Y., Chen Y., Hu H. (2023). *Bna.EPF2* Enhances Drought Tolerance by Regulating Stomatal Development and Stomatal Size in Brassica napus. Int. J. Mol. Sci..

[49] Fan M., Dorussen D., Gherli H., Lawson T. (2025). Reduced stomatal density in wheat overexpressing EPIDERMAL PATTERNING FACTOR1 differentially affects red and blue light responses. Plant Physiol..

[50] Guo T., Lu Z.Q., Xiong Y., Shan J.X., Ye W.W., Dong N.Q., Kan Y., Yang Y.B., Zhao H.Y., Yu H.X. (2023). Optimization of rice panicle architecture by specifically suppressing ligand-receptor pairs. Nat. Commun..

[51] Bai P., Torii K.U. (2026). ERECTA-family receptor kinases: Versatile regulators of plant developmental signaling. Plant J. Cell Mol. Biol..

[52] Hara K., Kajita R., Torii K.U., Bergmann D.C., Kakimoto T. (2007). The secretory peptide gene *EPF1* enforces the stomatal one-cell-spacing rule. Genes Dev..

[53] Melotto M., Underwood W., Koczan J., Nomura K., He S.Y. (2006). Plant stomata function in innate immunity against bacterial invasion. Cell.

[54] Doheny-Adams T., Hunt L., Franks P.J., Beerling D.J., Gray J.E. (2012). Genetic manipulation of stomatal density influences stomatal size, plant growth and tolerance to restricted water supply across a growth carbon dioxide gradient. Philos. Trans. R. Soc. Lond. Ser. B Biol. Sci..

[55] Ma X., Zhang Q., Zhu Q., Liu W., Chen Y., Qiu R., Wang B., Yang Z., Li H., Lin Y. (2015). A Robust CRISPR/Cas9 System for Convenient, High-Efficiency Multiplex Genome Editing in Monocot and Dicot Plants. Mol. Plant.

[56] Xie X., Ma X., Zhu Q., Zeng D., Li G., Liu Y.G. (2017). CRISPR-GE: A Convenient Software Toolkit for CRISPR-Based Genome Editing. Mol. Plant.

[57] Zhao H., Ma T., Wang X., Deng Y., Ma H., Zhang R., Zhao J. (2015). *OsAUX1* controls lateral root initiation in rice (*Oryza sativa* L.). Plant Cell Environ..

[58] Guo M., Zhao H., He Z., Zhang W., She Z., Mohammadi M.A., Shi C., Yan M., Tian D., Qin Y. (2022). Comparative Expression Profiling of Snf2 Family Genes During Reproductive Development and Stress Responses in Rice. Front. Plant Sci..

[59] Guo M., Zheng C., Shi C., Lu X., She Z., Jiang S., Tian D., Qin Y. (2025). The *OsZHD1* and *OsZHD2*, Two Zinc Finger Homeobox Transcription Factor, Redundantly Control Grain Size by Influencing Cell Proliferation in Rice. Rice.

[60] Cai H., Zhang M., Chai M., He Q., Huang X., Zhao L., Qin Y. (2019). Epigenetic regulation of anthocyanin biosynthesis by an antagonistic interaction between H2A.Z and H3K4me3. New Phytol..

[61] Jain M., Nijhawan A., Tyagi A.K., Khurana J.P. (2006). Validation of housekeeping genes as internal control for studying gene expression in rice by quantitative real-time PCR. Biochem. Biophys. Res. Commun..

[62] Schmittgen T.D., Livak K.J. (2008). Analyzing real-time PCR data by the comparative C(T) method. Nat. Protoc..

[63] Livak K.J., Schmittgen T.D. (2001). Analysis of relative gene expression data using real-time quantitative PCR and the 2(-Delta Delta C(T)) Method. Methods.

[64] Goodstein D.M., Shu S., Howson R., Neupane R., Hayes R.D., Fazo J., Mitros T., Dirks W., Hellsten U., Putnam N. (2012). Phytozome: A comparative platform for green plant genomics. Nucleic Acids Res..

[65] Chen C., Chen H., Zhang Y., Thomas H.R., Frank M.H., He Y., Xia R. (2020). TBtools: An Integrative Toolkit Developed for Interactive Analyses of Big Biological Data. Mol. Plant.

[66] Chen C., Wu Y., Li J., Wang X., Zeng Z., Xu J., Liu Y., Feng J., Chen H., He Y. (2023). TBtools-II: A “one for all, all for one” bioinformatics platform for biological big-data mining. Mol. Plant.

[67] Park C.H., Chen S., Shirsekar G., Zhou B., Khang C.H., Songkumarn P., Afzal A.J., Ning Y., Wang R., Bellizzi M. (2012). The Magnaporthe oryzae effector AvrPiz-t targets the RING E3 ubiquitin ligase APIP6 to suppress pathogen-associated molecular pattern-triggered immunity in rice. Plant Cell.

[68] Wang X., Diao Z., Cao C., Liu Y., Xia N., Zhang Y., Lu L., Kong F., Zhou H., Chen L. (2024). The receptor-like cytoplasmic kinase OsBSK1-2 regulates immunity via an HLH/bHLH complex. J. Integr. Plant Biol..

[69] Kachroo P., Leong S.A., Chattoo B.B. (1994). Pot2, an inverted repeat transposon from the rice blast fungus Magnaporthe grisea. Mol. Gen. Genet. MGG.

[70] Ke Y., Hui S., Yuan M.J.B.P. (2017). Xanthomonas oryzae pv. oryzae Inoculation and Growth Rate on Rice by Leaf Clipping Method. Bio-protocol.

[71] Wang C., Zhang X., Fan Y., Gao Y., Zhu Q., Zheng C., Qin T., Li Y., Che J., Zhang M. (2015). XA23 Is an Executor R Protein and Confers Broad-Spectrum Disease Resistance in Rice. Mol. Plant.

